# Establishing evidence-based decision-making mechanism in a health eco-system and its linkages with health service coverage in 25 high-priority districts of Uttar Pradesh, India

**DOI:** 10.1186/s12913-021-06172-2

**Published:** 2021-09-13

**Authors:** Ravi Prakash, Bidyadhar Dehury, Charu Yadav, Anand Bhushan Tripathi, Chhavi Sodhi, Huzaifa Bilal, N. Vasanthakumar, Shajy Isac, B. M. Ramesh, James Blanchard, Ties Boerma

**Affiliations:** 1 Department of Community Health Sciences, Institute of Global Public Health, University of Manitoba, R070 Med Rehab Bldg, 771 McDermot Avenue, Manitoba R3E 0T6 Winnipeg, Canada; 2 India Health Action Trust (IHAT), Lucknow, India

**Keywords:** Evidence-based review, Health management information system, India

## Abstract

**Background:**

Achievement of successful health outcomes depends on evidence-based programming and implementation of effective health interventions. Routine Health Management Information System is one of the most valuable data sets to support evidence-based programming, however, evidence on systemic use of routine monitoring data for problem-solving and improving health outcomes remain negligible. We attempt to understand the effects of systematic evidence-based review mechanism on improving health outcomes in Uttar Pradesh, India.

**Methods:**

Data comes from decision-tracking system and routine health management information system for period Nov-2017 to Mar-2019 covering 6963 health facilities across 25 high-priority districts of the state. Decision-tracking data captured pattern of decisions taken, actions planned and completed, while the latter one provided information on service coverage outcomes over time. Three service coverage indicators, namely, pregnant women receiving 4 or more times ANC and haemoglobin testing during pregnancy, delivered at the health facility, and receive post-partum care within 48 h of delivery were used as outcomes. Univariate and bivariate analyses were conducted.

**Results:**

Total 412 decisions were taken during the study reference period and a majority were related to ante-natal care services (31%) followed by delivery (16%) and post-natal services (16%). About 21% decisions-taken were focused on improving data quality. By 1 year, 67% of actions planned based on these decisions were completed, 26% were in progress, and the remaining 7% were not completed. We found that, over a year, districts witnessing > 20 percentage-point increase in outcomes were also the districts with significantly higher action completion rates (> 80%) compared to the districts with < 10 percentage-point increase in outcomes having completion of action plans around 50–70%.

**Conclusions:**

Findings revealed a significantly higher improvement in coverage outcomes among the districts which used routine health management data to conduct monthly review meetings and had high actions completion rates. A data-based review-mechanisms could specifically identify programmatic gaps in service delivery leading to strategic decision making by district authorities to bridge the programmatic gaps. Going forward, establishing systematic evidence-based review platforms can be an important strategy to improve health outcomes and promote the use of routine health monitoring system data in any setting.

**Supplementary Information:**

The online version contains supplementary material available at 10.1186/s12913-021-06172-2.

## Background

With more than 220 million estimated population in the year 2019, Uttar Pradesh (UP) is one of the most populous states in India. The state’s population contributes about one-sixth of the country’s population, with about 78% of people residing in rural areas [1]. Not only population-wise, but the state has various administrative layers (18 administrative divisions, 75 districts, 820 sub-district boundaries and more than 107,000 rural villages) which make the implementation of any health intervention very challenging [1]. Estimates from nationally representative surveys show that most of the key maternal and child health outcomes like maternal mortality ratio (MMR), infant, neonatal and under-five mortality rates and crude death rates are higher in the state compared to national estimates (Table S1) [2–4]. Therefore, the attainment of successful health outcomes in the state would largely depend on the effective implementation of health interventions supported by evidence-based programme planning and implementation.

Availability of routine and quality data is pivotal for evidence-based programming. Similar to other national and international contexts, the Government of Uttar Pradesh (GoUP) implemented routine Health Management Information System (HMIS) in 2009 [5] that enables effective decision making by providing health care data at different levels. This provided a basis to analyse the multiple critical health system functions required for planning, coordination and implementation of health programs [6]. Globally, the routine HMIS data has witnessed various limitations which inhibit health programmes from utilising them to make appropriate decisions. Consequently, this has led to a greater inclination towards the use of survey data while taking any strategic decisions. The issues around completeness, quality and coverage of HMIS data are well documented [7–10]. Most of them are also applicable to India and the state of UP as well. Results from independent studies conducted in Uttar Pradesh have identified key constraints affecting data use for decision-making, including a lack of clear processes for data collection and analysis; little agreement on key performance indicators among stakeholders within the health system; a lack of incentives to promote data use; and limited data analysis skills among health staff [11, 12]. Moreover, HMIS data largely comprises of output indicators while information on input and process indicators are almost missing.

In October 2013, through an independent funding from Bill and Melinda Gates Foundation (BMGF), the University of Manitoba and India Health Action Trust established an Uttar Pradesh Technical Support Unit (UP TSU) to support the GoUP in enhancing the efficiency and effectiveness of implementing the national reproductive, maternal, neonatal, child health (RMNCH) programme in the state. The intervention area of the UP TSU was primarily concentrated in the 25 high-priority districts (HPDs) of the state against total 75 districts. These HPDs ranked the lowest in the performance of maternal and child health outcomes compared to the rest of the state, which formed the basis of their selection into this category (Table S2). These 25 districts also served as a platform for UP TSU to implement and test various intervention strategies before scaling-up to the state level. Some of the key interventions included improving the availability, quality and utilization of ANC services through establishing service delivery platforms for ANC, integration of front line workers (FLWs) work by strengthening AAA (ASHA, Anganwadi worker, ANM) platform, assisting them with the supportive supervision and appropriate job aids, focusing on high-risk pregnancies, and building the counselling skills of FLWs. The community work also focused on improving post-natal care by emphasizing upon the improvisation of home-based newborn care visits and integrating family planning and nutrition-related counselling during those visits. On the facility fronts, efforts were made to activate delivery points to enhance institutional delivery, improve quality of intrapartum and immediate postpartum care, activate first referral units and mentoring of doctors, and institutionalise nurse mentors at block level/sub-centre facilities to provide quality services at lower-level facilities. Along with the community and facility level interventions, strengthening the availability and quality of HMIS data to enhance data use was one of the key levers of UP TSU’s intervention under the health system strengthening platform. Recognising the limitations of HMIS, at the outset, UP TSU advocated with the GoUP to establish an integrated digital platform, known as the Uttar Pradesh HMIS (UP-HMIS) with the objectives of (1) capturing missing input and process data required to develop programming; (2) providing relevant data to decentralised decision-makers to holistically measure the performance of health programmes; and (3) integrating different government data portals and manual reports of different health programmes into one centralized and electronic data portal – the UP-HMIS- for meaningful data analysis [13]. Also, a specific UP Health Dashboard was in-built within the UP-HMIS which ranks blocks and districts on a key set of 12 priority health indicators and 2 data quality indicators (Table S3). These rankings were meant to identify low performing and high performing blocks based on their performance on key indicators. Once the gap in performance was identified, further drill-down analysis within blocks helped to ascertain gaps in service delivery (e.g., availability of commodities, drugs, human resources) against the target. This then forms the basis of developing action plans depending on the identified reasons and framing potential solutions.

### The intervention: strengthening evidence-based review platforms

Even though UP TSU’s efforts helped the state in improving data availability and its’ quality to a large extent, the systematic use of data for problem-solving remained a challenge. For example, the availability of UP-HMIS data formats increased from 48% in 2014 to over 90% in 2016 and over 95% in 2020 (data not shown). The increase in data availability has contributed to improvements in other data quality metrics as well. For example, timely reporting from health facilities to UP-HMIS increased from 12% in 2017 to 94% by 2019. Similarly, there has been an increase in the number of reporting units (heath facilities) from 49% in 2014 to 98% in 2019 [Meghani A, Tripathi A, Bilal H, Gupta S, Prakash R, Namasivayam V et al., Optimizing the health management information system in Uttar Pradesh, India: Implementation insights and learnings, unpublished]. Similarly, on data quality, there has been a tremendous reduction in reporting of missing values (45 to 9% across different levels of health facilities). Outlier values for key programme review indicators also decreased from 7 to 4% for the indicator measuring third antenatal care check-up, 7 to 2% for institutional delivery, and 9 to 4% for full immunization of infants between the ages of 9–11 months indicating improved data quality.

Establishing a platform for data-based review was envisaged as one of the interventions to bring systematic use of data for problem-solving and bring the culture of data use in the government system. It was hypothesized that conducting evidence-based review meetings will develop the habit of data use among the health officials and thereby will not only contribute to improvements in data quality but also would lead to an improvement in health outcomes due to strategic decisions being taken based upon the review sessions. To do this, UP TSU helped GoUP in strengthening the existing review platform known as Monthly Medical-Officer-In-Charge Review Meetings (MMRM) in which sub-district level health program performances were reviewed by the district leadership on monthly basis. Although districts were conducting the review meetings, these meetings were largely based on manual data reported from the districts with limited authenticity. These review systems were also plagued by other issues, such as a) no uniform framework for the review meeting and data use, b) more focus on process than outcomes, c) focus on financial progress than programmatic, and d) no tracking mechanism for previously taken decisions.

With the new intervention, strengthening evidence-based review platforms, the system of review meeting was revised and efforts were made to strengthen MMRM bringing specific focus on reviewing the programme performance on coverage of several RMNCH services using a standard set of indicators and a prescribed analysis plan. A deep-dive analysis, available on the Health Dashboard, was considered as the starting point for identifying performance gaps. They were used to conduct review meetings and the decisions made in the meetings were tracked to ensure that district and block level health managers fulfilled their accountability in ensuring the completion of actions planned. These MMRMs not only provide an established platform for reviewing programme performance through robust data but also strengthen the use of these routinely validated data at block and district levels. A framework summarizing the key steps and guidelines released by the GoUP on implementing the program review meetings at the district level is presented in Figure S1. Considering MMRM as an important intervention to improve programmatic performance through establishing evidence-based review mechanism, the present paper attempts to understand the pattern of decisions taken during the MMRM, completion of action plans and its’ linkages with key service coverage indicators. In other words, it aims to understand as to how data use can improve the data quality and ultimately form the basis for improving health outcomes in a population. Findings from this study can be useful in advocating the establishment of a robust platform for the data-driven review of programme performance to enhance the culture of data use in the government system that subsequently improves the health outcomes.

## Methods

**Fig. 1:**
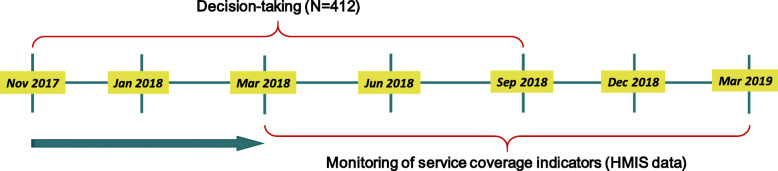
Timelines of two data sets used for analysis

Two data sets were used for analysis: i) the decision-tracking data collected from November 2017 to September 2018 and ii) health outcome/output data gathered between April 2018 to March 2019 reported on HMIS. The present analysis focuses on 25 HPDs due to the initial focus of the intervention in these geographies. Given the fact that it usually takes 2 to 3 months to complete an action for any kind of decision and another 3 months or so for that action to register any effect on population health outcomes, the decision-tracking and HMIS data were considered for different periods. A total of 412 decisions were taken by district officials between November 2017–September 2018 and these formed the basis for this analysis. Figure 1 provides a pictorial representation of the timeline for the analysis, along with data sources.

### Outcome variables

Three service coverage indicators, namely, the proportion of pregnant women with 4 or more ANC visits and received 4 or more haemoglobin tests (denoted as 4 + ANC and 4 + Hb) against estimated pregnant women, the proportion of institutional deliveries against estimated delivery, and women who had a post-natal check-up within 48 h of delivery were considered as outcomes. The aggregate district-level data from HMIS on these indicators were used for outcome analysis. Data from about 6963 healthcare facilities spanning 25 HPDs have been used herein.

### Analysis

The analyses involved three steps. The first step included conducting univariate analysis to understand the distribution of decisions-taken during the reference period by a few characteristics. Subsequently, the number of decisions taken versus the actions completed at the overall level was compared and stratified by the time. The relatively small size of the sample at the district level for some of the decision categories inhibited further stratification by districts. For the last set of analysis, we compared the decision-completion/ action-taken status across the districts which noticed a minimum improvement in the outcomes (< 10%-point increase), moderate improvement (10–20%-point increase) and maximum improvement (> 20%-point increase). Cut-off of 10, 20% and more than 20% was chosen based on the total change observed in the indicator values across 25 HPDs. While linking the action completed with the service coverage outcomes, only those decisions were taken into consideration that was relevant to the particular outcome. For example, while assessing the ANC and Haemoglobin (Hb) testing indicators, only those actions completed were taken into consideration, which was directly linked to either improving ANC coverage and/or Hb testing rather than those which were taken to improve institutional delivery or post-natal care (PNC). Univariate analysis was used to understand the distribution of decisions taken and actions completed whereas bi-variate analysis to understand the associations between actions taken in different domains and the three health outcomes. A *P-*value of < 0.05 was considered significant in bivariate analysis. Analyses were conducted in STATA 14.0.

## Results

Table 1 shows that, of the 412 decisions taken, about two-thirds were from the MNCH domain (31% ANC, 16% each on delivery and PNC, 8% each on child care and family planning, and 21% were related to data quality. A large district-level variation in the number of decisions taken and actions completed was observed. For instance, about 10–11% decisions were taken in the districts of Rampur and Shahjahanpur, followed by 7% in Budaun, 6% in Balrampur and Kannauj and 5% in the districts of Bareilly, Farrukhabad, and Kheri (Table 1). During the reference period, 67% of the planned actions were completed, 26% were ongoing or in progress, while the remaining 7 % could not be completed. While about 70% of all the planned actions were completed in ANC, PNC and data quality domains, the action completion rate was 53% for delivery, 58% for child care and only 45% for family planning.

**Table 1:** Profile of decision taken/action planned and completion status

Total decision taken and action planned	412
**Per cent distribution of action taken by domains**	
ANC	31.3
Delivery	16.3
PNC	16.0
Child care	7.5
Family Planning	7.5
Data quality	21.4
**Per cent distribution of action taken by districts**	
Rampur	10.9
Shahjahanpur	10.4
Budaun	7.0
Balrampur	5.6
Kannauj	5.6
Bareilly	5.1
Farrukhabad	4.9
Kaushambi	4.9
Kheri	4.6
Gonda	4.4
Barabanki	3.6
Mahrajganj	3.6
Shrawasti	3.6
Sonbhadra	3.6
Allahabad	3.4
Bahraich	2.9
Kanshiram Nagar	2.9
Hardoi	2.7
Sitapur	2.4
Pilibhit	1.9
Sant Kabir Nagar	1.7
Faizabad	1.2
Mirzapur	1.2
Siddharthnagar	1.2
Etah	0.5
**Per cent distribution of decisions/action by completion status**	
Completed	66.5
In-complete	26.2
Not done	7.2
**Percentage of action completed by domains**	
ANC	73.6 (129)
Delivery	56.7 (67)
PNC	72.7 (66)
Child care	58.1 (31)
Family Planning	45.2 (31)
Data quality	69.3 (88)

Number in parenthesis shows the number of actions taken in respective domains

**Fig. 2:**
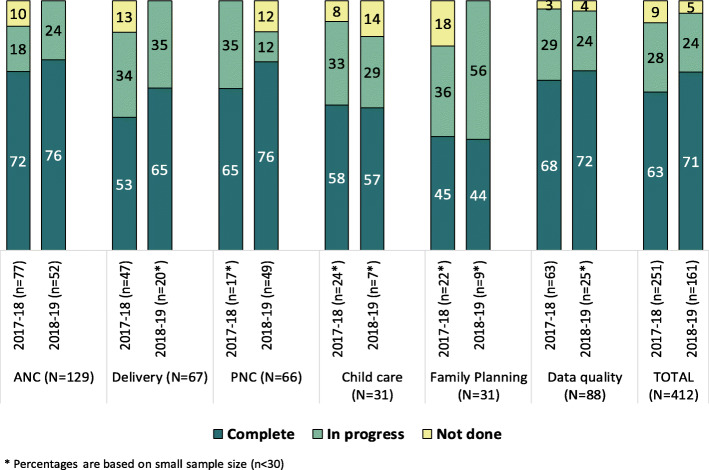
Percent distribution of action completion by domains and time period

Table 2 shows the proportion of decisions taken over the year and the corresponding actions by their completion status. It was observed that 60% of decisions were taken between Nov’17-Mar’18 and 40% between Apr’18-Sep’18 spanning over two financial years. While the pattern of these decisions was the same across the year in different programmatic domains- a significantly higher proportion of decisions in the PNC domain were taken in the last 6 months. The overall completion rate was almost similar for both the financial years (63% vs 71%) and the domains of child care (~ 58%) and family planning (~ 45%). The action completion rate increased in the domains of delivery (53 to 65%), PNC (65 to 76%), and data quality (68 to 72%), whereas remained almost same in the realm of ANC (72 to 76%) (Fig. 2).

**Table 2:** Percentage of action plan developed between Nov’17-Sep’18 by programme domains

Programme domain	Nov’17-Mar’18 (%)	Apr’18-Sep’18 (%)
ANC	60.0	40.0
Delivery	70.0	30.0
PNC	26.0	74.0
Child care	77.0	23.0
Family Planning	71.0	29.0
Data quality	72.0	28.0
Total	61.0	39.0

**Fig. 3:**
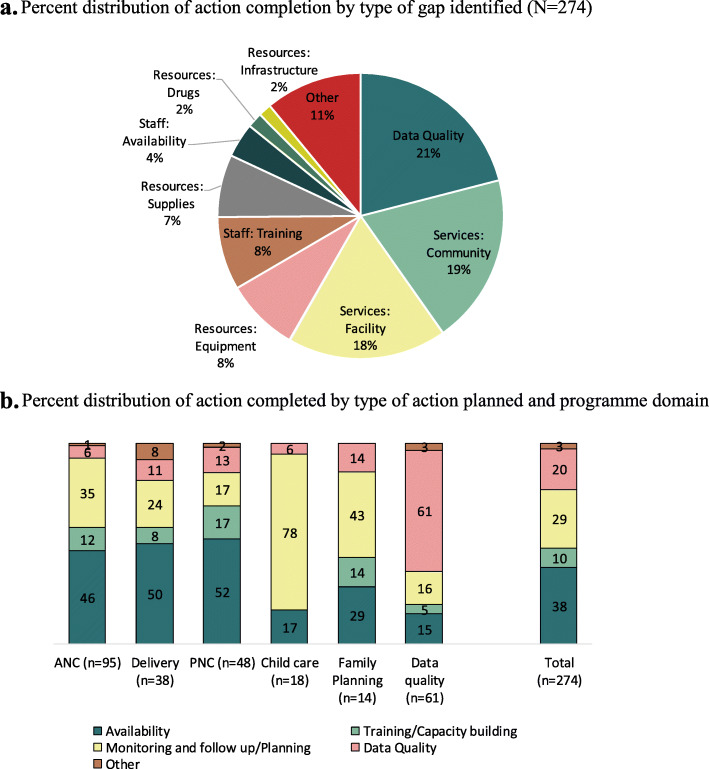
**a** Percent distribution of action completion by type of gap identified (*N* = 274). **b** Percent distribution of action completed by type of action planned and programme domain

While we analysed the action plan completion status by programmatic domains, it was also important to categorise these actions into the types of programmatic gaps identified and actions taken. Five major gap areas were identified, which include 1) availability of human resources (HR), infrastructure and equipment; 2) training or capacity building to improve the performance and quality of services; 3) monitoring and follow up / planning to improve service provisioning or utilization; 4) data quality - to improve or strengthen data availability as well as data quality; 5) others. All the action completed were also categorised corresponding to the identified gaps. Figure 3a depicts that about two-thirds to the identified gaps were related to community and facility-related service delivery and skilled HR, about 21% were linked to data quality and 10% were related to the availability of resources such as equipment and adequate infrastructure. Staff availability and training also appeared to be important domains which affected programme performance and formed the focus of substantial attention from the district-level programme managers.

Figure 3b represents the completion status of actions planned in the different programme domains. Overall, of the 274 completed actions, a majority of them were related to availability (38%), followed by monitoring and follow-up/planning (29%), data quality (20%) and 10% were related to training or capacity building. About half of the completed action plans that were accomplished within the domains of ANC, delivery and PNC were related to the availability of HR, infrastructure or equipment, 17 and 35% were related to monitoring and follow-up/ planning in the domain of ANC and delivery, respectively. A total of 6–13% were related to data quality, training or capacity building and monitoring and follow-up/ planning in the domain of PNC. In the domains of child care and family planning, a greater part of the action plans was related to monitoring (78%) and follow-up/planning (43%).

### Association between action completion and service coverage outcomes

In the bivariate analysis, the proportion of actions completed were assessed in groups of districts according to the level of changes in health outcomes. Table 3 shows the level of service coverage among the different group of districts categorised based on their improvement in service coverage indicators - minimum (< 10% points), medium (10–20% points), and maximum (> 20% points) over the period. The districts with minimum improvement in 4 + ANC and 4 + Hb varied from 50 to 53% between Apr-Jun, 2018 and Jan-Mar, 2019; whereas the recorded change in coverage extended from 45 to 60% and 44 to 73%, respectively, for districts with medium and maximum improvements. Similar patterns were also observed for the other two indicators on institutional delivery and postnatal care within 48 h of birth.

**Table 3:** Improvements in service coverage indicators during the first and last quarter of the financial year 2018–19, HMIS

Indicators	Levels of service coverage indicators (%)								
	Minimum improvement (< 10% point increase)			Medium improvement (10–20% point increase)			Maximum improvement (> 20% point increase)		
	*Apr-Jun 2018*	*Jan-Mar 2019*	*# of districts*	*Apr-Jun 2018*	*Jan-Mar 2019*	*# of districts*	*Apr-Jun 2018*	*Jan-Mar 2019*	*# of districts*
4^+^ ANC and 4^+^ Hb	50%	53%	7	45%	60%	10	44%	73%	8
Institutional delivery	46%	54%	8	48%	61%	9	45%	63%	8
PNC within 48 h	48%	44%	10	36%	51%	9	36%	74%	6

For assessing whether the group of districts which logged minimum to moderate improvements in service coverage indicators were also the districts with a fewer proportion of action plans completed vis-a-vis districts with maximum gains in service coverage indicators, we analysed the action plan completion rate of these three distinct groups of districts. Table 4 depicts that the action completion (related to ANC) was 56 and 71% in the districts which recorded minimum and medium improvement in the outcomes, while it stood at 86% in the districts which had a maximum improvement in outcome indicators (*p* = 0.001). Similarly, the percentage of actions completed was higher in the districts with maximum improvement in institutional delivery and PNC within 48 h of delivery compared to the districts with minimum improvement (institutional delivery: 84% vs 66%; *p* = 0.016) PNC within 48 h: 81% vs 68%; *p* = 0.339).

**Table 4:** Changes in health outcomes and action completion in 25 HPDs, UP

Indicators	Action plans completed (%)				
	Minimum improvement (< 10% point increase)	Medium improvement (10–20% point increase)	Maximum improvement (> 20% point increase)	Minimum vs medium improvement	Minimum vs maximum improvement
	*Nov 2017-* *Dec 2018*	*Nov 2017-* *Dec 2018*	*Nov 2017-* *Dec 2018*	*P-value*	*P-value*
4^+^ ANC and 4^+^ Hb	56% (34)	71% (38)	86% (57)	0.186	**0.001**
Institutional delivery	54% (79)	66% (56)	84% (61)	0.159	**0.016**
PNC within 48 h	68% (34)	75% (16)	81% (16)	0.613	0.339

## Discussion

The evidence-based MMRM appears to be an important intervention for improving health outcomes in the 25 HPDs. Our study suggests that districts with improvement in coverage outcomes were also the districts where a large proportion of planned actions were completed. It also indicates that continuous monitoring of the actions planned, based on the identified gaps and ensuring the completion of planned activities may contribute to significant changes in health outcomes. Not only that, using available data, districts were also able to identify the correct programmatic gaps and could take appropriate decisions to fill in the gaps. Previous literature has argued that management skills – such as strategic problem solving, human resource management, financial management and operations management – are fundamental to health service system strengthening [14, 15]. Our findings suggest that once the district-management starts taking decisions and actions on correctly identified gaps in programmatic and management related issues, it can devise appropriate strategies to improve health outcomes.

The study results show that there were a few districts where many decisions and action completion were taken while a bunch of districts with lesser decisions or action completion. This again shows that any intervention takes time to graduate and saturate in any setting. It also depends on the pro-activeness of the district leadership. Thus, the districts having more decisions taken and action completion may also depend on the level of activeness of district leadership and their sensitiveness towards evidence-based programming. This also gets reflected in the findings that the higher levels of the decision taken and action completion was observed in the second half of the financial year compared to the first half. This could also be attributed to the fact that once the district started realising the importance of having an evidence-based reviewed meeting, close monitoring of decision-made and action completion on improvement in health outcomes, they would have started commencing such meetings more regularly. Furthermore, the in-built data analysis packages under the UP Health dashboard coupled with state-level close monitoring of the monthly performance of districts and blocks helped in gearing up the intervention in many districts.

The present analysis has a few limitations as well. First, the analysis is based on decision-tracker data. There were a few decisions for which either action completion status/date was not mentioned or the specific action warranted in lieu of certain decisions was not reported. Hence, the analysis was restricted to only those decisions for which complete information was available on all specified parameters. Second, the analysis relates to the changes in coverage of the decisions. There might be other programmatic factors that may have contributed to the improvement in coverage indicators. Also, there is a possibility that for a similar type of programmatic gap, different intensity of decisions were made across different districts and may have differential effects on the findings. Since the solution of an identified gap varies by the geography and context, it could not be standardized. Third, the present analysis is bi-variate in nature and does not offer any opportunity to ascertain the causality between MMRM decision tracker and the improvement in outcomes. Moreover, since the intervention was initially focused on 25 HPDs, data on decision tracking is not available from the remaining 50 districts of the state. This posed a restriction in evaluating the ‘true effect’ of intervention on outcomes. Also, the availability of data for a fewer number of districts constringed us from venturing into the further deep-dive analysis. The analysis did not take into account the role of supportive supervision visits in ensuring the implementation of decisions/actions in the district due to the unavailability of any robust data in this regard. Lastly, information on decision making and actions before the implementation of the MMRM would have also been beneficial.

Despite its limitations, the study is one of its kind globally which attempted to understand the effect of strengthening the data-based review platform on health outcomes in any setting. Numerous studies have earlier focused on establishing improvements in health outcomes through better health management practices utilising facility-level data, demonstrating that practical education and mentoring in management can promote significant improvements in the quality and consistency of health service delivery [16–27]. They are, however, deficient in establishing a relationship between district-level health management and health service system performance in terms of enhancing the coverage of service delivery. Thus, while these studies were important from the health management perspective, they did not focus much on understanding how the data-based decisions at the district level translated into improvements in health outcomes. The former forms an important contextual layer within the larger healthcare management system. Using district-level data, which encompasses information for healthcare facilities at different levels of functioning, this study attempted to fill in this information gap and offers the possibility of generalizing the findings to other healthcare settings.

## Conclusion

Overall, the support of UP TSU to the government of UP in developing UP-HMIS and inculcating the culture of data-based review mechanism is the first step towards ‘centralizing’ the health data in the state and is a part of GoUP’s broader vision to develop a comprehensive and integrated digital government health data portal. The long-term vision is to develop an integrated information communication technology architecture that would allow the flow of health data across different data portals into one central dashboard repository that visually presents key programme monitoring data to support programme management and decision making at various levels in the health system 19. Moreover, using the data for reviewing the programme performance, not just in terms of financial outflow, but for tracking the improvement in coverage of beneficiaries for a range of health service interventions will be one of the potential ways to achieve the sustainable development goals of achieving a reduction in maternal and neonatal mortality in the state.

## Supplementary Information

**Additional file 1: Table S1.** Key maternal and child health outcomes, India and Uttar Pradesh.

**Additional file 2: Table S2.** Key maternal and child health outcomes by HPD, non-HPD, State.

**Additional file 3: Table S3.** List of 14 indicators used in UP Health Dashboard for district and block ranking.

**Additional file 4: Table S4.** Status of three coverage indicators and % change over time across 25 HPDs.

**Additional file 5: Figure S1.** Process of enhancing the use of data for decision making during program review meetings at the district level.

## Data Availability

The datasets generated and/or analysed during the current study are available in the Government of India repository and can be assessed at https://hmis.nhp.gov.in/#!/. The decision-tracking data is not available in the public domain, however available from the corresponding author on reasonable request.

## Abbreviations

ANC: Ante-natal Care
ANM: Auxiliary Nurse Midwife
ASHA: Accredited Social Health Activist
GoUP: Government of Uttar Pradesh
Hb: Haemoglobin
HMIS: Health Management Information System
HPD: High Priority District
HR: Human Resource
MMR: Maternal Mortality Ratio
MMRM: Monthly Medical officer-in-charge Review Meeting
PNC: Post-natal Care
RMNCH: Reproductive, Maternal, Neonatal and Child Health
UP HMIS: Uttar Pradesh Health Management Information System
UP TSU: Uttar Pradesh Technical Support Unit
UP: Uttar Pradesh
