# Supplementary material for: Establishing evidence-based decision-making mechanism in a health eco-system and its linkages with health service coverage in 25 high-priority districts of Uttar Pradesh, India
Source: BMC Health Serv Res. 2021 Sep 13;21(Suppl 1):196. doi: 10.1186/s12913-021-06172-2 (PMC8436494; doi:10.1186/s12913-021-06172-2)
Supplement: Supplementary file 1 — Additional file 1: Table S1. Key maternal and child health outcomes, India and Uttar Pradesh. [file 12913_2021_6172_MOESM1_ESM.docx]

**Table S1:** Key maternal and child health outcomes, India and Uttar Pradesh

| Indicator | Uttar Pradesh | India | Data Source (Year) |
| --- | --- | --- | --- |
| Maternal mortality ratio | 197 | 113 | Sample Registration System (2016-18) |
| Infant mortality rate | 43 | 32 | Sample Registration System (2018) |
| Neonatal mortality rate | 32 | 23 | Sample Registration System (2018) |
| Under-5 mortality rate | 47 | 36 | Sample Registration System (2018) |
| Crude birth rate | 25.6 | 20.0 | Sample Registration System (2018) |
| Total fertility rate | 2.7 | 2.2 | National Family Health Survey (2015-16) |
