# Supplementary material for: Establishing evidence-based decision-making mechanism in a health eco-system and its linkages with health service coverage in 25 high-priority districts of Uttar Pradesh, India
Source: BMC Health Serv Res. 2021 Sep 13;21(Suppl 1):196. doi: 10.1186/s12913-021-06172-2 (PMC8436494; doi:10.1186/s12913-021-06172-2)
Supplement: Supplementary file 2 — Additional file 2: Table S2. Key maternal and child health outcomes by HPD, non-HPD, State. [file 12913_2021_6172_MOESM2_ESM.docx]

**Table S2:** Key maternal and child health outcomes by HPD, non-HPD, State

| Indicator | HPD | Non-HPD | Uttar Pradesh |
| --- | --- | --- | --- |
| Neonatal mortality rate per 1000 live births | 55.9 | 44.0 | 49.0 |
| Infant mortality rate per 1000 live births | 73.3 | 61.6 | 68.0 |
| Safe delivery (%) | 58.7 | 70.9 | 63.3 |
| Full-immunization (%) | 45.0 | 58.0 | 52.7 |
| Modern contraceptive prevalence rate | 30.8 | 40.9 | 37.6 |
| Total fertility rate | 3.9 | 3.1 | 3.3 |
| Source: Annual Health Survey; 2012-13 |  |  |  |
