# Supplementary material for: Establishing evidence-based decision-making mechanism in a health eco-system and its linkages with health service coverage in 25 high-priority districts of Uttar Pradesh, India
Source: BMC Health Serv Res. 2021 Sep 13;21(Suppl 1):196. doi: 10.1186/s12913-021-06172-2 (PMC8436494; doi:10.1186/s12913-021-06172-2)
Supplement: Supplementary file 3 — Additional file 3: Table S3. List of 14 indicators used in UP Health Dashboard for district and block ranking. [file 12913_2021_6172_MOESM3_ESM.docx]

**Table S3:** List of 14 indicators used in UP Health Dashboard for district and block ranking

| # | Domain | Indicator | Type | Source |
| --- | --- | --- | --- | --- |
| 1 | Ante-Natal | % of pregnant women received 4 or more ANC and tested for Hb against estimated PW |  | UPHMIS |
| *1A* |  | *% of pregnant women received 4 or more ANC against estimated PW* | *Coverage* | UPHMIS |
| *1B* |  | *% of pregnant women tested for Hb for 4 or more times against estimated PW* | *Quality* | UPHMIS |
| 2 | Delivery care | % of pregnant women delivered in an institution against an estimated delivery | Coverage | UPHMIS |
| 3 |  | % of C-section delivery against reported delivery (70% weightage to CHC and 30% to DH) | Quality | UPHMIS |
| 4 |  | Stillbirth ratio | Quality | UPHMIS |
| 5 | Post-natal care | % of women receiving a postpartum check within 48 hours of home delivery against reported delivery (Home +institutional)* | Coverage | UPHMIS |
| 6 | Immunization | Ratio of Pentavalent 3 to BCG | Quality | UPHMIS |
| 7 |  | % of children received full immunization | Coverage | RCH |
| 8 | Family Planning | % of eligible couple accepted limiting method | Coverage | UPHMIS |
| 9 |  | % of eligible couple accepted spacing method | Coverage | UPHMIS |
| 10 | Communicable Disease | Total case notification rate of TB against expected TB cases | Quality | RNTCP MIS |
| 11 |  | % of PW screened for HIV against estimated pregnancy | Coverage | UPHMIS |
| 12 | Fund utilization | Per ASHA expenditure of ASHA incentive fund | Coverage | BCPM MIS |
| 13 | Data quality: Completeness | % of units reported non-blank value (including zero) for the identified indicators of the ranking | Data quality | UPHMIS |
| 14 | Data quality: Consistency | % of units reported outlier for the identified indicators of the ranking | Data quality | UPHMIS |
