# Supplementary material for: Establishing evidence-based decision-making mechanism in a health eco-system and its linkages with health service coverage in 25 high-priority districts of Uttar Pradesh, India
Source: BMC Health Serv Res. 2021 Sep 13;21(Suppl 1):196. doi: 10.1186/s12913-021-06172-2 (PMC8436494; doi:10.1186/s12913-021-06172-2)
Supplement: Supplementary file 4 — Additional file 4: Table S4. Status of three coverage indicators and % change over time across 25 HPDs. [file 12913_2021_6172_MOESM4_ESM.docx]

**Table S4:** Status of three coverage indicators and % change over time across 25 HPDs

| **Indicators** | **Sep-Nov, 2017** | **Sep-Nov, 2018** | **Sep-Nov, 2019** | **% change (2017 to 2018)** | **% change (2018 to 2019)** | **% change (2017 to 2019)** |
| --- | --- | --- | --- | --- | --- | --- |
| 4^+^ ANC and 4^+^ Hb | 48.0 | 59.0 | 79.7 | 22.9 | 35.1 | 66.0 |
| Institutional delivery | 49.6 | 50.7 | 58.6 | 2.2 | 15.5 | 18.0 |
| PNC within 48 hrs | 42.5 | 48.0 | 76.3 | 12.9 | 59.0 | 79.6 |

*Source: HMIS*
