# Supplementary material for: Establishing evidence-based decision-making mechanism in a health eco-system and its linkages with health service coverage in 25 high-priority districts of Uttar Pradesh, India
Source: BMC Health Serv Res. 2021 Sep 13;21(Suppl 1):196. doi: 10.1186/s12913-021-06172-2 (PMC8436494; doi:10.1186/s12913-021-06172-2)
Supplement: Supplementary file 5 — Additional file 5: Figure S1. Process of enhancing the use of data for decision making during program review meetings at the district level. [file 12913_2021_6172_MOESM5_ESM.docx]

**Figure S1.** Process of enhancing the use of data for decision making during program review meetings at the district level

**
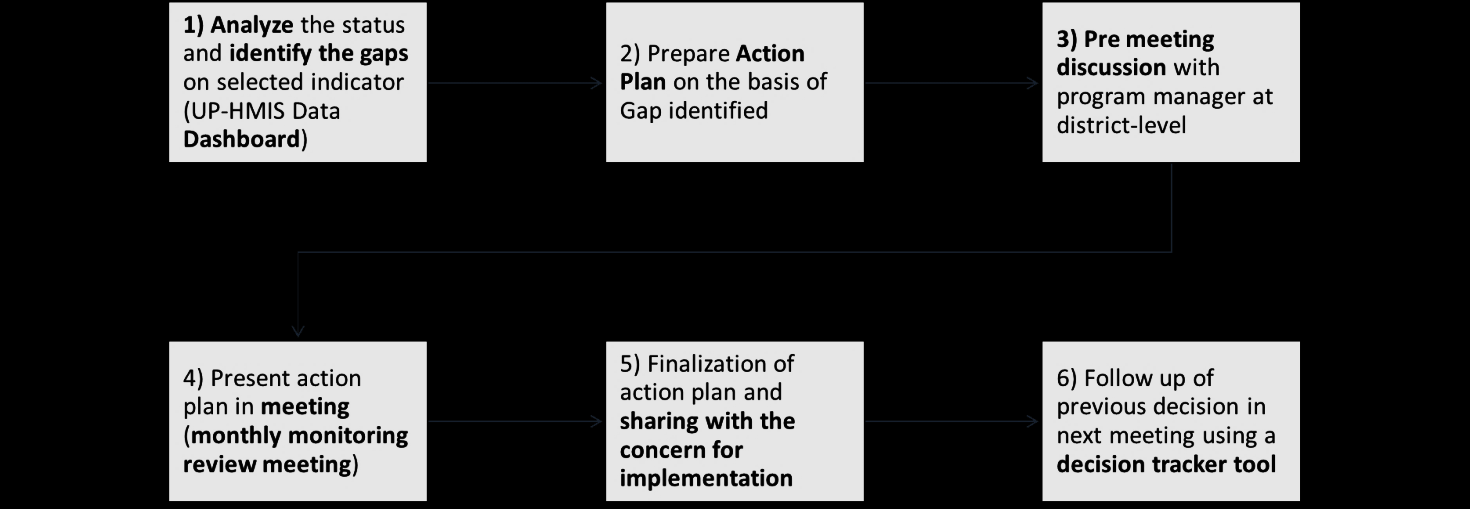
**
